# Infantile TK2 Deficiency Causing Mitochondrial Encephalomyopathy With Migrating Focal Seizures

**DOI:** 10.1212/WNL.0000000000213373

**Published:** 2025-03-03

**Authors:** Luca Bergonzini, Sara Carli, Silvia Pelle, Ilaria Pettenuzzo, Silvia Bonetti, Erika Santi, Caterina Visconti, Monica Maffei, Marta Sheremet, Eleonora Lamantea, Andrea Marsala, Olena Klub, Valentina Gentile, Duccio Maria Cordelli, Caterina Garone

**Affiliations:** 1 Department of Medical and Surgical Sciences, Alma Mater Studiorum University of Bologna, Italy;; 2 IRCCS Istituto delle Scienze Neurologiche di Bologna, U.O.C. Neuropsichiatria dell'età pediatrica, Italy;; 3 IRCCS Istituto delle Scienze Neurologiche di Bologna, Programma di neuroradiologia con tecniche ad elevata complessità, Bologna, Italy;; 4 Pediatric Department, Western Ukrainian Specialized Children's Medical Center, Lviv, Ukraine; and; 5 Unit of Medical Genetics and Neurogenetics, Fondazione IRCCS Istituto Neurologico Carlo Besta, Milan, Italy.

## Abstract

**Objective:**

Recessive variants in the *TK2* gene cause thymidine kinase 2 deficiency (TK2d) presenting with infantile, childhood, or adult-onset myopathy. CNS involvement is reported in only 25% of the infantile form. Compassionate use of deoxynucleoside substrate enhancement therapy (dC/dT) has been demonstrated safe and effective in TK2d myopathy, but no data are available on the potential efficacy on the human brain disease.

**Methods:**

Here, we report for the first time a patient with infantile TK2d epileptic encephalomyopathy enrolled in an early access program with dC/dT treatment (MT1621).

**Results:**

At age 3 months, he presented progressive hypotonia, motor regression, failure to thrive, and respiratory failure. At age 8 months, he developed drug-resistant epilepsy with migrating focal seizures. Brain MRI showed progressive atrophy and bilateral subcortical lesions with lactate peak. Exome sequencing revealed 2 novel biallelic heterozygous variants in the *TK2* gene (c.182G>A, p.Ser61Asn, c.704 T>C, p.Ile235Thr) whose pathogenicity was confirmed with in vitro studies. Early access compassionate use of dC/dT at 400 mg/kg prolonged the survival and stabilized the muscle disease but was not effective on the brain.

**Discussion:**

Our report highlights the importance of deep-phenotyping infantile TK2d before dC/dT supplementation to stratify disease severity further and suggests a limited tissue-specific brain efficacy.

## Introduction

Autosomal recessive variants in the *TK2* gene cause a lack of enzyme activity (thymidine kinase 2 deficiency, TK2d), nucleotide pool unbalance, and consequently depletion and/or multiple deletions of mitochondrial DNA (mtDNA). TK2d is classified into infantile, childhood, and adult myopathy with the highest morbidity and mortality for the early-onset cases.^1^ CNS involvement has been reported in a limited number of cases with infantile TK2d (eTable 1).^1^

Preclinical in vivo and in vitro models have demonstrated the efficacy of nucleos(t)ides supplementation therapies (dC/dT) in TK2d.^2,3^ Treatment has already been translated under compassionate use or controlled clinical trials into human use and demonstrated safe and effective in case series of pediatric and adult patients with myopathy,^4,5^ but no data are available on CNS efficacy in humans.

Here, we report the clinical, imaging, molecular genetics, and biochemical data of a 16-month-old infant with TK2d epileptic encephalomyopathy treated with dC/dT (MT1621).

## Methods

MT1621 early access program was approved by the ethical committee of our institution, and the informed consent was obtained according to the Declaration of Helsinki. Methods for in vitro studies are available in eMethods.

### Data Availability

The anonymized data can be accessed on reasonable request addressed to the corresponding author.

### Statistical Analysis

GraphPad 8 was used for statistical analysis. A D'Agostino-Pearson normality test was used to evaluate the normal distribution of data, whereas an unpaired Student t-test to compare groups. Data were represented as mean ± SEM and statistical significance as: *p < 0.05, **p < 0.01, ***p < 0.001 and ****p < 0.0001.

#### Case Description

The patient is the fourth child of 2 healthy, nonconsanguineous Ukrainian parents. It was reported that an older brother died at the age of 9 months with myopathy and respiratory insufficiency. The proband was born by spontaneous delivery after an uneventful pregnancy. He presented developmental regression, progressive axial and peripheral hypotonia, muscle weakness, ptosis, feeding difficulties, and vomiting at age 3 months. His Children's Hospital of Philadelphia Infant Test of Neuromuscular Disorders score progressively dropped from 58/64 to 7/64 points by the age of 7 months. He lost the ability to make vocal sounds but maintained eye contact. Electroneurography showed bilateral axonal neuronal injury. A percutaneous endoscopic gastrostomy and a tracheostomy were performed because of progressive worsening of the feeding difficulties, failure to thrive, and respiratory muscle failure. Whole exome sequencing analysis showed 2 heterozygous missense variants of uncertain significance (VUS) in the *TK2* gene (NM_004614.5) in exons 3 (c.182G>A, p.Ser61Asn) and 10 (c.704T>C, p.Ile235Thr). He was admitted to our hospital when he was 8 months old to confirm the diagnosis and enroll him in an early access program with MT1621. Laboratory analysis showed increased CK (688 U/L, normal value (n.v.) <145) and transaminase (AST 146 U/L, n.v. <60, ALT 95 U/L, n.v. <45) levels. He also presented with hepatomegaly and obstructive concentric hypertrophic cardiomyopathy with normal systolic function at ultrasound studies. Muscle MRI showed generalized muscle atrophy, with adipose infiltration, most prominent in the gluteal, quadriceps, and soleus muscles.

**Figure 1:**
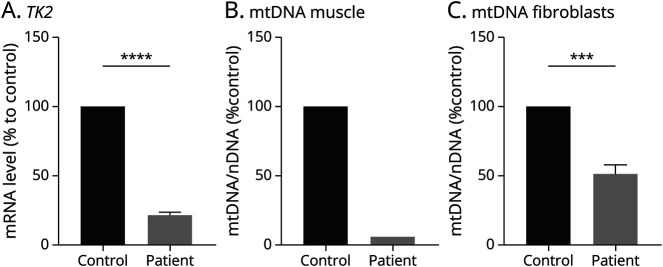
Functional Studies Confirming the Pathogenicity of the TK2 Gene Variants (A) *TK2* gene expression revealing severe reduction in the patient (n = 2 biological replicates) compared with healthy control (n = 2 biological replicates), (B and C) mtDNA copy number in DNA extracted from muscle homogenates (B) and fibroblast cell line (C) showing severe reduction in the patient (n = 1 biological replicates in muscle, 2 in fibroblast) compared with the healthy control (CTRL, n = 1 biological replicates in muscle, 2 in fibroblast). Data derived from at least 3 independent experiments. All data are expressed as a percentage of control and presented as mean ± SEM. ***p < 0.001, ****p < 0.0001.

**Figure 2:**
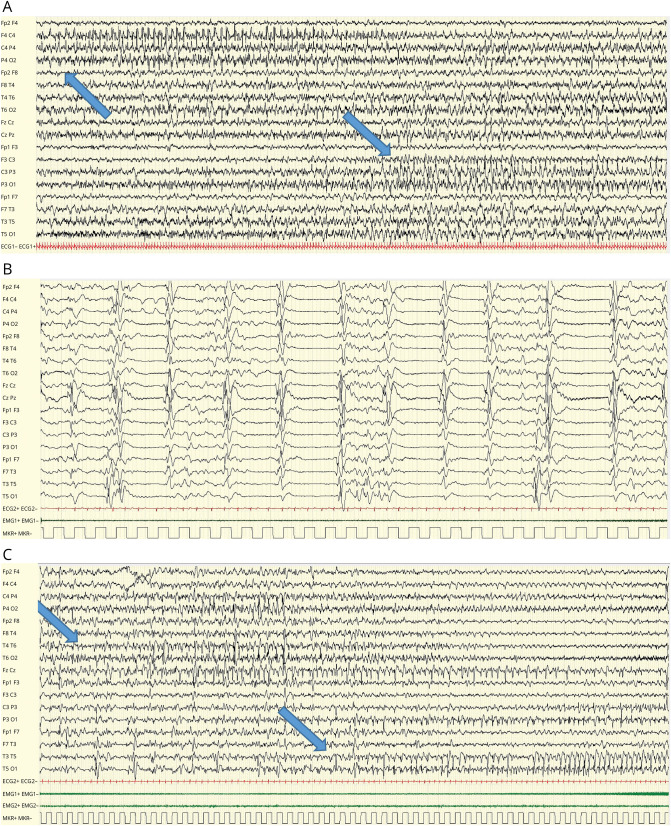
EEG Pattern During Continuous Video-EEG Monitoring (A) Ictal EEG at 8 months showing focal seizures migrating from the right to the left hemisphere (black arrows, 90 seconds/page), (B and C) follow-up at age 10 months showing progressive EEG worsening with disruption of background activity, burst suppression pattern (B: 30 seconds/page), and subcontinuous migrating focal seizures (black arrows, C: 60 seconds/page).

**Figure 3:**
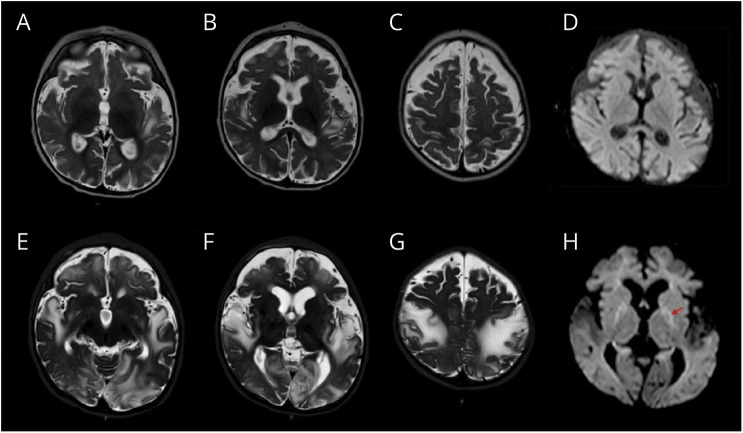
Brain MRI Study (A–D) T2-weighted images showing high-intensity signal in the insular and postcentral gyrus areas and sub/cortical atrophy at 8 months, (E–H) follow-up study at 10 months showing severe worsening of signal alterations over time, with spread to the basal ganglia, temporo-occipital and frontal regions and progression of the sub/cortical atrophy over time.

Sanger sequence analysis confirmed the presence of the 2 biallelic heterozygous variants and their segregation in the parents (c.182G>A maternal allele, c.704T>C paternal allele). Downregulation of *TK2* mRNA expression was found in the patient's fibroblasts (Figure 1A), but the sequencing of the whole cDNA did not identify additional rearrangements. Mitochondrial respiratory chain activities in muscle biopsy showed multiple OXPHOS deficiencies (eTable 2). Quantitative real-time PCR in muscle and fibroblasts' DNA showed severe mtDNA depletion confirming the pathogenicity of the variants (Figure 1, B and C) and modifying their classification from VUS to pathogenic. Treatment with MT1621 was started and progressively titrated to the target dose of 400 mg/kg/d for each deoxynucleoside. Initially, motor function improvement was observed: he was able to partially control his head and move from side to side with eye contact. CK and transaminase gradually normalized. Both hepatomegaly and hypertrophic cardiomyopathy were stable over time. However, we observed paroxysmal episodes of subtle fine, stereotyped, clonic movements involving the mouth, fingers, and toes, contralaterally migrating within the same event, and episodes with flushing or desaturation. A 24-hour video EEG recorded seizures and showed focal-onset monomorphic, rhythmic, sharp, theta activity in the bilateral temporoparietal regions, with a migrating propagation pattern from one hemisphere to the other, correlating with the clinical semiology (Figure 2A). Brain MRI identified 2 symmetric, cortical, and subcortical T2-weighted hyperintense signal areas in the insular and postcentral regions (Figure 3, A–D). The clinical and EEG features were suggestive of epilepsy of infancy with migrating focal seizures (EIMFS). Several antiseizure medications (i.e.,: levetiracetam, carbamazepine, phenobarbital, rufinamide, clobazam, clonazepam) and ketogenic diet at a 4:1 ratio were ineffective in reducing seizure frequency. Follow-up EEG showed background activity disruption, progressively leading to a suppression-burst pattern and subcontinuous focal seizure onset progressing to status epilepticus (Figure 2, B and C). CNS functions globally worsened with respiratory drive loss and the necessity of continuous ventilation. Follow-up brain MRI showed progressively diffuse brain atrophy with bilateral T2 hyperintense cortical and subcortical lesions spreading to the temporo-occipital and frontal regions and evolving to malacic areas by age 10 months. MRI spectroscopy confirmed the presence of lactic acid peaks (eFigure 1). Contrarily, muscle MRI showed a stable pattern with no progression of muscle atrophy. Treatment with MT1621 was continued for its partial efficacy on the patient's general condition, but the patient died at 18 months from multiorgan failure after an episode of viral gastroenteritis.

## Discussion

Tk2d causes an ultra-rare mitochondrial myopathy.^1^ Our report is the first description of infantile-onset migrating seizures occurring in TK2d. Continuous video-EEG monitoring was crucial to highlight the migrating pattern of focal seizures that were initially clinically subtle, being in the context of severe generalized myopathy. Migrating focal seizures with no underlying structural causal lesions are the distinctive feature of EIMFS, a rare, severe developmental and epileptic encephalopathy with drug-resistant epilepsy, developmental impairment, and regression. EIMFS is a genetically heterogeneous syndrome with at least 35 disease-causing genes identified, encoding for proteins responsible for neuronal excitability and synaptic functions^6-9^ or associated with metabolic disorders.^6,9,10^ Our report suggests including the *TK2* gene in the genetic screening of EIMFS-like disorders.

CNS involvement has been reported in a subgroup of 12 patients with infantile TK2d manifesting with seizures and/or cognitive impairment within 1 to 18 months from the onset of myopathy. No genotype-phenotype correlation, nor other disease mechanism has been identified explaining the brain disease in this subgroup of patients. Neuroimaging and clinical follow-up data are limited in literature with no distinctive abnormalities reported at brain MRI.^1,11,12^ In our case, serial brain MRI showed rapidly progressive diffuse brain atrophy with bilateral gray and white matter lesions evolving to malacic areas that were predictive of a poor prognosis.

Preclinical studies in the *Tk2* H126N knock-in mouse model demonstrated the safety and dose-dependent systemic efficacy of nucleos(t)ide therapy including the ability to cross the blood-brain barrier and ameliorate brain phenotype.^2,3^ Our report demonstrated that nucleoside therapy stabilized muscle disease but did not prevent the progression of CNS involvement. Although we cannot exclude that an earlier intervention could have better efficacy, these results suggest that brain involvement is a negative prognostic factor for MT1621 efficacy. Further studies in a larger cohort of patients are needed to confirm our observation and give definitive guidelines.

In conclusion, TK2d can cause severe epileptic encephalomyopathy in a subgroup of infantile-onset patients. Deep phenotyping including brain MRI and EEG studies is essential to further stratify the disease phenotype and severity in infantile Tk2d, evaluate the potential benefit and indication for treatment, and give complete informative to the family.
